# MOET Efficiency in a Spanish Herd of Japanese Black Heifers and Analysis of Environmental and Metabolic Determinants

**DOI:** 10.3390/biology11020225

**Published:** 2022-01-30

**Authors:** Juan M. Vázquez-Mosquera, Aitor Fernández-Novo, Martin Bonet-Bo, Natividad Pérez-Villalobos, Jose L. Pesántez-Pacheco, Maria Luz Pérez-Solana, Eduardo de Mercado, Juan Carlos Gardón, Arantxa Villagrá, Francisco Sebastián, Sonia Salomé Pérez-Garnelo, Daniel Martínez, Susana Astiz

**Affiliations:** 1 Medicine and Surgery Department, Veterinary Faculty, Complutense University of Madrid, Puerta de Hierro Avenue s/n, 28040 Madrid, Spain; juvazq01@ucm.es; 2 Department of Veterinary Medicine, School of Biomedical and Health Sciences, Universidad Europea de Madrid, C/Tajo s/n, 28670 Villaviciosa de Odón, Spain; aitor.fernandez@universidadeuropea.es (A.F.-N.); natividad.perez@universidadeuropea.es (N.P.-V.); 3 Embriovet SL, Polígono Industrial de Piadela II-8, A Coruña, 15300 Betanzos, Spain; martin.bonet@embriovet.es (M.B.-B.); daniel@embriovet.es (D.M.); 4 Faculty of Agricultural Sciences, School of Veterinary Medicine and Zootechnics, University of Cuenca, Av. Doce de Octubre, Cuenca 010220, Ecuador; jose.pesantez@ucuenca.edu.ec; 5 Animal Reproduction Department, National Institute of Agronomic Research (INIA-CSIC), Puerta de Hierro Avenue s/n, 28040 Madrid, Spain; luz.perez@inia.es (M.L.P.-S.); eduardo.mercado@inia.es (E.d.M.); sgarnelo@inia.es (S.S.P.-G.); 6 Department of Animal Medicine and Surgery, Veterinary and Experimental Sciences School, Catholic University of Valencia-San Vicente Mártir, Guillem de Castro, 94, 46001 Valencia, Spain; jc.gardon@ucv.es; 7 Institut Valencià d’Investigacions Agràries (IVIA), CV-315, Km, 10700 Valencia, Spain; villagra_ara@gva.es; 8 Cowvet SL, Avda. País Valenciano 6, Betera, 46117 Valencia, Spain; cowvetsl@gmail.com

**Keywords:** donor, recipients, management, cholesterol, Japanese Black

## Abstract

**Simple Summary:**

Embryo transfer procedures have been widely implemented in bovine all around the world. These techniques help to accelerate the increase in the genetic merit and to keep the level of inbreeding under control, which is especially important in breeds, such as Japanese Black cattle, in areas outside of Japan, where there are scarce individuals and few herds. Our study describes an adequate embryo productivity of Japanese Black heifers under Spanish management and environmental conditions, like that which has been previously reported, demonstrating the adequate adaptation capacity of these animals. No effect on embryo production or quality was found due to plasma metabolic parameters of the donors, suggesting an optimal nutritional and body conditioning of the donors’ herd. Pregnant recipients after embryo transfer showed significantly higher levels of cholesterol-related parameters, glucose, and urea, which may be related to higher availability of nutrients for the future pregnancy. Heat stress at embryo transfer negatively impacted conception rates as expected, while larger parity and ET number corresponded to numerically higher conception rates.

**Abstract:**

Multiple ovulation and embryo transfer (MOET) systems have been intensively implemented in Japanese Black cattle in Japan and to create Japanese Black herds out of these areas. Environmental conditions influence MOET efficiency. Thus, we describe results of 137 in vivo, non-surgical embryo flushings performed between 2016–2020, in a full-blood Japanese Black herd kept in Spain and the possible effects of heat, year, bull, donor genetic value, and metabolic condition. Additionally, 687 embryo transfers were studied for conception rate (CR) and recipient related factors. A total of 71.3% of viable embryos (724/1015) were obtained (5.3 ± 4.34/flushing). Donor metabolites did not affect embryo production (*p* > 0.1), although metabolite differences were observed over the years, and by flushing order, probably related to the donor age. CR was not affected by embryo type (fresh vs. frozen), recipient breed, and whether suckling or not suckling (*p* > 0.1). CR decreased significantly with heat (44.3 vs. 49.2%; (*p* = 0.042)) and numerically increased with recipient parity and ET-number. Pregnant recipients showed significantly higher levels of cholesterol-related metabolites, glucose, and urea (*p* < 0.05). Therefore, adequate MOET efficiency can be achieved under these conditions, and heat stress should be strongly avoided during Japanese Black embryo transfers. Moreover, recipients’ metabolites are important to achieve pregnancy, being probably related to better nutrient availability during pregnancy.

## 1. Introduction

The most common beef cattle breed in Japan is Japanese Black, also known as Wagyu, which derives from native Asian cattle. There are Japanese Black herds in Australia and North America that export animals, semen, and embryos [1,2]. The Japanese Black breed has different characteristics from other bovine breeds, such as higher oleic acid concentration in intramuscular fat (52.9% of oleic acid in subcutaneous fat) [3,4], which lead to different beef aroma and palatability, as well as a high level of marbling or intramuscular fat content (IMF), ultimately translating to meat of high market value [2]. The IMF in dry matter of Japanese Black cattle at 24 months old is 23.3% [5]. The greater marbling rate reflects metabolic features of Japanese Black purebreds: insulin concentration in plasma correlates positively with carcass fat and negatively with carcass muscle [6], and Japanese Black steers have higher insulin levels and lower glucose levels in plasma than Holstein steers [7]. Japanese Black cattle also show higher levels of cholesterol, phospho-lipids and metabolites, such as urea, than Holstein cattle, but similar levels of albumin, triglycerides, and non-esterified fatty acids (NEFA) [7].

Japanese Black differ from other bovine breeds not only in biochemical features but also in other parameters linked to adipose tissue, such as leptin levels. The protein leptin is an adipocyte-derived circulating hormone that signals the body to decrease food intake, which can halt body weight gain [8]. Leptin also regulates the expression of some genes and the activation of certain enzymes [9,10]. Polymorphisms in the leptin gene are associated with economically important carcass traits [11,12,13,14], milk production traits [15,16], fertility traits [17], and immune function [18,19]. Leptin levels in Japanese Black cattle normally lie around 2.67 ± 0.41 ng/mL [20], while levels in Holstein cattle have been reported to be around 0.3–0.55 nmol/L [21] or 1.79 ± 0.43 ng/mL [20].

Reproductive biotechnologies, which have been widely used for decades in beef cattle to optimize birth rates and herd genetics [22,23], have played an important role in reducing inbreeding of Japanese Black cattle in Japan and elsewhere [24]. These techniques can quickly generate highly valuable purebred herds [1,25] and include freezing semen from selected bulls, artificial insemination (AI), embryo collection, and “multiple ovulation and embryo transfer” (MOET) of fresh or frozen–thawed embryos.

Little is known about the reproductive efficiency of Japanese Black cattle. One study reported a mean parity of 4.9 ± 2.9, a mean interval from calving to first service of 80.0 ± 46.2 days, and a first service conception rate (CR) of 53.5 ± 49.9% [26]. Another study [27] described an average CR after AI of 47.0% in heifers and 47.8% in adult cows, with first, second, and third AIs leading to higher CR values than fourth or subsequent AIs. Higher CRs have been reported for other beef breeds in Europe, such as 59.3% [28] or 63.6% [29] for Angus, 63.6% for Charolais, 63.2% for Limousine, and 56.9% for cross-breeds [30]. Embryo transfer appears to work reasonably well in the Japanese Black breed: in one study, 685 oocytes were collected from 8 multiparous Japanese Black cows and 120 embryos were achieved after in vitro fertilization (IVF), and transferring 36 such embryos into Holstein heifers led to 20 pregnancies [31]. Another study reported similar pregnancy rates around 50% during embryo transfer from Japanese Black into Holsteins [24], achieving similar results for single or twin transfer of frozen–thawed embryos.

Reproductive efficiency of Japanese Black cattle appears to have decreased since 1990 [32]. This may reflect the cost of genetic programs prioritizing meat quality over other characteristics [33]. Since meat quality is closely related to metabolism, optimizing meat quality may influence reproductive performance. Several factors have been identified that affect reproductive efficiency after MOET and other reproductive biotechnologies. These factors include stress, nutrition, disease, and biosecurity [34,35,36,37]; environmental temperature, asynchrony of estrous cycle between donors and recipients, and luteal insufficiency [38]; and progesterone concentrations in plasma [38,39]. How these factors interact with metabolism to influence reproductive efficiency in the Japanese Black cattle is unclear.

We aimed to investigate environmental, management, hormonal, and metabolic factors affecting MOET reproductive efficiency of Japanese Black heifers. We analyzed a full-blood Japanese Black herd in Spain that was subjected to MOET for five years. The data from this herd provide an important extension to the literature, much of which centers on herds in Japan. Reproductive traits of Japanese Black heifers are strongly influenced by farm management practices and the farm environment [40], which can differ substantially between Japanese and Spanish farms. Japanese traditional management is intensive and emphasizes limiting animal movement, even in reproductive herds [26], in order to optimize fat infiltration and meat quality [33]. In contrast, the Spanish farm in our study emphasized animal welfare and therefore provided each heifer with a relatively large surface area and access to the natural environment. The typical Japanese climate is warm and features humid summers and cold winters [26], in contrast to Spain’s hot, dry summers and mild winters. In Japan, cattle are typically fed barley, corn and soybean, supplemented with high fiber products such as okra, nut husks and grasses and hay, and often, rice and wheat straw; in contrast to local cereals with olive and orange waste products on our Spanish farm [41].

Our hypothesis was that the different management and environmental conditions on our Spanish farm would affect MOET performance by altering the physiological and metabolism of the Japanese Black cattle.

## 2. Material and Methods

### 2.1. Animals and Herd

The study was performed on the commercial farm “Mudéjar-Wagyu” in north-central Spain (40°37′42.1″ N 0°41′08.0″ W, Aliaga, Teruel). This herd was managed under an intensive MOET program from 2016 through 2020. Data on flushings were analyzed for the period 2016–2020, while data on embryo transfers were analyzed for 2018–2020.

During the study period, maximal temperatures in Aliaga occurred during the summer (July–August), with the average maximum being 26 °C. Minimal temperatures occurred in winter (December–February), with the average minimum being −2 °C. The highest maximal temperature was 33.4 in July and the lowest minimal temperature was −10.9 °C in January. Rainfall was strong in spring (April–May) and autumn (mainly October), and maximal annual rainfall was 34–35 mm. January was the driest month (7 mm), but it featured the highest snowfall (41 mm). Snow persisted between November and March, when the monthly snowfall averaged 25 mm. Average maximal humidity was 35% and ranged from 71.2% in March to 0.4% in June [42,43,44].

All flushings and transfers were part of routine veterinary work, so no additional interventions were performed during this study that could be considered experimental procedures. Therefore, no ethical approval was required as stipulated in the Spanish Policy for Animal Protection (RD 53/2013), which complies with the European Union Directive 2010/63/UE on the protection of research animals.

On the study farm, three separate barns were used for the breeding herd, defined as donor cows and recipient cow–calf pairs; growing animals, defined as those after weaning until 22 months of age; and animals in the last phase of fattening, defined as those older than 22 months until slaughter. All barns were open with natural ventilation and had anti-slip concrete floors covered with chopped straw bedding. Cows in barns had access to the outside until two weeks before calving, when they were moved to the maternity barn. Calves remained with their mothers until weaning at 3–5 months of age, during which time they could move over to a shaded surface area of 20 m^2^. Cows had ad libitum access to water and a total mixed ration (TMR) feed that was adjusted to their age and physiological stage (pregnant or suckling) [45]. Calves had ad libitum access to starter food until weaning.

Genetic consultants classified donors into one of three groups depending on their phenotypic characteristics and the genetic value of their predecessors: high value, referred to below as “type A”; intermediate value, referred to below as “type B”; or “ordinary”. The study herd included 33 type A donors, for which we collected data on 109 flushings: 6 heifers were flushed once, 11 twice, 6 three times, 3 four times, 1 five times, 3 six times, 1 seven times, 1 ten times, and 1 eleven times. The herd included three type B heifers, for which we collected data on 13 flushings: 1 heifer was flushed three times, 1 twice, and 1 eight times. Lastly, the herd included 3 ordinary donors, for which we collected data on 15 flushings: 1 heifer was flushed three times, 1 four times, and 1 eight times. Donors that were found not to have produced any high-quality embryos were not included in subsequent flushings. The intended interval between flushings was 45 d.

Recipients and donors underwent routine health examinations as well as general and reproductive examinations in order to be included in synchronization protocols for embryo transfer or superovulation. Recipient animals came from a herd of different breeds (55 dams/year). These cows were selected for their special maternal characteristics and were subjected to repeated embryo transfers after at least 6 months postpartum. Recipient animals were excluded from the study if they had more than two unsuccessful embryo transfers in the same cycle, a history of abortion, or a history of rejection of the previous calf. A total of 287 Holstein recipients on three Holstein farms different from the study farm were used, and the same inclusion and exclusion criteria and management practices as for the recipients’ herd on the studied farm were applied. Data for 687 embryo transfers were included in the study.

### 2.2. Donor Synchronization and Superovulation

**Figure 1:**
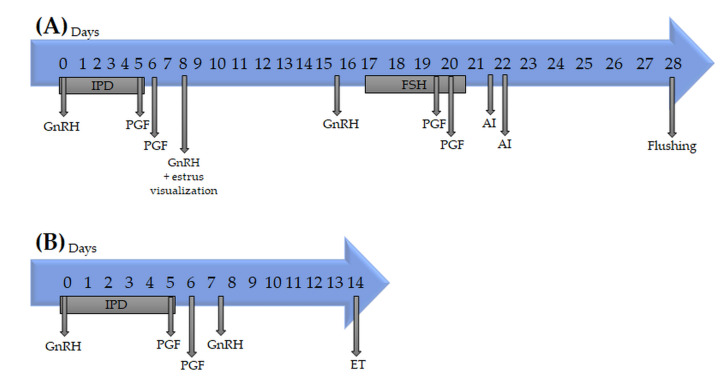
Hormonal protocols for superovulation and flushing in donors (**A**) and for embryo transfer in recipients (**B**). Abbreviations: IPD = intravaginal progesterone device; PGF = prostaglandin; GnRH = gonadorelin; FSH = follicle stimulant hormone; AI = artificial insemination; ET = embryo transfer.

Every cycling donor was submitted to estrus synchronization based on a 5–day Ovsynch protocol and an intravaginal progesterone device (Figure 1). In brief, the protocol consisted of intramuscular administration of 100 μg GnRH (Cystoreline*^®^*, CEVA Santé Animale SA, Libourne, France) and insertion of a 1.55 g intravaginal progesterone device (PRID-delta*^®^*, CEVA Santé Animale, Libourne, France) on Day 0. On Day 5, 0.5 mg cloprostenol (Bioestrovet*^®^*, Vetoquinol SA, Lure, France) was administered intramuscularly, and the progesterone device was removed. On Day 6, 0.5 mg cloprostenol was administered. Heat detection patches (Estrotect^TM^, Estrotec GmbH, Düsseldorf, Germany) were placed in all donors at this time.

On Day 8, 100 μg GnRH (Cystoreline*^®^*) was administered to induce ovulation, and heat signs were recorded based on visual inspection of patches during Days 8 and 9. On Days 15–16 (7 days after heat detection), an ultrasound examination was conducted (IBEX Pro-Ei Medical, CO, USA). Animals with an apparently functional corpus luteum and a dominant follicle with a diameter >0.8 mm received a double intramuscular dose (200 μg) of GnRH (Cystoreline*^®^*).

At 36–48 h after GnRH injection on Days 15–16, follicle-stimulating hormone (FSH; Folltropin-V*^®^*, Vetoquinol SA, Lure, France) was administered twice daily (8 a.m. and 8 p.m.) on Days 9 to 12 (days 24 to 28 of the protocol) to give a total dose of 240 mg. The doses were decreased eight times as follows: Day 9, 50 mg each at a.m. and p.m.; Day 10, 40 mg at a.m. and 30 mg at p.m.; Day 11, 30 mg at a.m. and 20 mg at p.m.; and Day 12, 20 mg at a.m. and 10 mg at p.m.

Simultaneously, with the sixth and seventh FSH administrations on Days 27 and 28 of the protocol, luteolysis was induced using two doses of cloprostenol, 12 h appart (0.5 mg per dose; Bioestrovet*^®^*). At 50 and 72 h after the first cloprostenol administration, two fixed-time artificial inseminations (FTAIs) were performed, with one dose per FTAI of conventional Japanese Black frozen–thawed semen from 12 purebred Japanese Black bulls of high genetic quality that had been controlled for inbreeding.

### 2.3. Flushing of Embryos and Their Evaluation

Data were included on 137 non-surgical embryo flushings on Japanese Black heifers aged 21.8 ± 6.79 months. Embryos were collected by the authorized embryo producer Embriovet SL (A Coruña, Spain; EU license ES11ET05B) using the “open method”. This method involved silicone catheters (14–16 gauge) with two-way Luer locks one ends, 50-mL syringes and epidural anesthesia (120 mg Pronestesic*^®^* procaine; Fatro S.p.A., Bologna, Italy). Each horn was flushed with 240 mL of flushing medium (BoviFlush*^®^*; Minitube, Tiefenbach, Germany) through 8 inflow–outflow cycles, and the recovered fluid was filtered through a 75 µm mesh (emSafe, Minitube).

Prostaglandin was administered after flushing and estrus was observed, as expected, 4–7 days later. Natural estrus was observed an average of 21 days later, and on the ninth day after natural estrus, the superovulation procedure was repeated. With this system, an objective between flushing intervals of 45 days was intended in heifers flushed repeatedly.

Immediately after each flushing, embryos were assessed under a stereoscope (Leica S APO-10, Leica Microsystems, Bucharest, Romania) at 10× magnification within a dish containing holding medium (ViGRO^TM^ Holding Plus, Vetoquinol). We evaluated embryos at 80× magnification, and viable embryos were washed 10 times in 300 µL drops of holding medium (ViGRO^TM^ Holding Plus), assessed for development stage and quality according to IETS guidelines [46], individually labeled, and placed into 0.25 mL sterile straws (IMV, France).

Fresh embryos graded as 1–3 were transferred to selected recipients. Only grade 1 surplus embryos were frozen. Freezing was performed according to a conventional slow freezing protocol for direct transfer in an automatic freezer (Freeze Control*^®^* CL5500, CryoLogic, Victoria, Australia). This procotol involved 10 min of equilibration at room temperature in ethylene glycol (BoviFreeze*^®^*; Minitüb GmbH Tiefenbach, Germany), seeding and holding at −6 °C for 10 min, then cooling at 0.5 °C per min down to −35 °C. Finally, straws were plunged into liquid nitrogen [47] and later used for direct, one-step embryo transfer [48].

### 2.4. Recipient Synchronization and Embryo Transfer

A total of 687 embryo transfers were performed over the years of the study (2018–2020), using a total of 11 Brown Swiss, 63 Simmental, 11 Charolais, and 23 crossbred recipient cows located at the same farm of the donors (the Mudéjar-Wagyu-farm). These cows aged 54.9 ± 25.80 months old at the moment of ET. Additionally, further ETs were performed at commercial dairy cattle farms. In these farms, only Holstein heifers were included, which aged 13.3 ± 5.80 months.

Cycling, healthy recipients (heifers and post–calving cows) were synchronized using the same 5–day Co–synch with intravaginal progesterone device explained above. Briefly (Figure 1): on Day 0, administration was carried out with 100 μg GnRH i.m. (Cystoreline*^®^*, CEVA) and insertion of a 1.55 g IPD (PRID-delta*^®^*, CEVA Santé Animale, Libourne, France); on day 5 and 6, 0.5 mg cloprostenol were administered i.m. (Bioestrovet*^®^*, Vetoquinol); and on Day 6, a heat detection patch (ESTROTECT^TM^) was placed; on Day 8, 100 μg GnRH i.m. (Cystoreline*^®^* CEVA) was applied to induce ovulation, and heat signs were recorded using hip patches. Seven days after estrus detection recipients were examined by ultrasound exam (IBEX Pro- Ei medical, Loveland, CO, USA) confirming the absence of genital, uterine, and ovarian pathologies, and the presence of a corpus luteum. The ovary with the corpus luteum was recorded. Epidural anesthesia was performed with 120 mg of procaine (Pronestesic*^®^*; Fatro) before each ipsilateral embryo transfer. The synchronization protocol and examinations of the recipients at external Holstein heifers were the same as described above. In these heifers, only frozen–thawed embryos were transferred.

From the total of 687 transferred embryos, 270 fresh and 277 frozen embryos were obtained from the studied Japanese Black donors previously described, while the additional 140 were frozen imported embryos (Blackmore Japanese Black, Alexandra, Victoria, Australia).

A pregnancy diagnosis was performed 30–35 days after ET by an experienced veterinarian by ultrasound scanner (IBEX Pro-Ei medical, Colorado, CO, USA). Pregnancy confirmation was performed on days 120–130 of gestation.2.5.

Metabolite analyses assessments were performed in blood plasma samples obtained by coccygeal vein puncture once on one of the first 5 days of the synchronization protocol, with a random 50% of the donors (76/137), and on the day of pregnancy diagnosis performed 30–35 days after ET, on 30% of the recipients (246/687), with standard 10 mL ethylene diamine tetra-acetic acid (EDTA) vacuum tubes (Vacutainer*^®^* System Europe; Becton Dickinson, Meylan, France). Blood samples were centrifuged at 4500× *g* for 15 min, and the plasma was separated and stored in polypropylene vials at −80 °C until later assayed with a clinical chemistry analyzer (Konelab 20; Thermo Scientific, Thermo Fisher Scientific, Waltham, MA, USA) according to the manufacturer’s instructions. Beta-hydroxybutyrate (BHB, mmol/L), non-esterified-fatty acid (NEFA mmol/L), plasma total cholesterol (TC, mg/L), high density lipoprotein cholesterol (HDL, mg/L), low density lipoprotein cholesterol (LDL, mg/L), glucose (GLU, mg/L), lactate (LAC, mg/L), triglycerides (TG, mg/L), urea (UR, mg/dL) and fructosamine (FRU, mg/L) were measured. Plasma leptin concentrations (LEP, ng/mL) were also determined by using a multispecies ELISA kit (Linco Research Inc., St. Charles, MO, USA; assay sensitivity 1.0 ng/mL and intra-assay variation coefficient 3.1%).

### 2.5. Statistical Analysis

All data were analyzed using SPSS**^®^** 25 (IBM, Armonk, NY, USA) except for the normality test for all variables, which was the Shapiro–Francia test, only available at STATA**^®^** software V. 17 (StataCorp LLC, Lakeway Drive, Texas, TX, USA). Variables showing a skewed distribution were reported as median and range instead of average and standard deviation as best measures of centrality and variability for skewed distributions; intergroup differences in these variables were assessed for significance using a Kruskal–Wallis test or Mann–Whitney U test for independent samples. Potential pairwise relationships between donor characteristics and metabolic traits and embryo transfer outcomes were explored using Pearson correlation.

Logistic regression was used to assess effects of different factors on the probability of pregnancy after embryo transfer. In this process, stepwise forward modeling based on the Wald statistic criterion of *p* > 0.10 was used. The model included embryo type, embryo origin, embryo quality, recipient breed, body condition score (BCS), parity, open days, heat stress at embryo transfer, suckling status at embryo transfer, as well as pairwise interactions between these effects. Recipient and farm were included as fixed factors.

For all analyses, statistical significance was defined at a 5% threshold (*p* < 0.05), while tendencies were defined at *p* < 0.10.

## 3. Results

### 3.1. Embryo Flushings and Donor Metabolite Analyses

A total of 137 flushings, performed from 2016 to 2020 at the Mudéjar-Wagyu farm, were included in the study. The Japanese Black animals aged 21.8 ± 6.79 months (range 12 to 4 months old) were flushed; average amount of flushings per heifer was 3.27 (range from 1 to 11 times), with the average interval between flushings being 73 ± 40 days (range 41 to 238 days). Body condition score (BCS) average was 3.5 ± 0.57 (BCS measured 1 to 5) [49].

Embryo flushings were distributed over time (2016–2020) as follows: 10 flushings in 2016 (7.3%, 10/137), 26 in 2017 (19.0%, 26/137), 36 in 2018 (26.3%, 36/137), 38 in 2019 (27.7%, 38/137), and 27 in 2020 (19.7%, 27/137). From these 137 flushings, 102 were performed under no heat stress (74.5%, 102/137; from 16 September to 14 June), and 35 under heat stress conditions (25.5%, 35/137; from 15 June to 15 September). Most of the animals (75.2%) were flushed one to four times, and the rest (24.8%) were flushed more than four times.

A total of 1015 embryos were produced from these 137 flushings with an average of 7.4 ± 5.18 embryos per collection. Viable embryos were 724 (71.3%; 5.3 ± 4.34 viable embryos per flushing), 165 degenerated embryos (1.3 ± 1.56/flushing), and 126 non-fertilized oocytes. From these 724 viable embryos, 440 were freshly transferred and 284 embryos were frozen. From these embryos, a total of 270 fresh embryos and a total of 277 frozen–thawed embryos were transferred since 2018 and included in the transfers study (described below).

We found no statistically significant effect of the donor genetic line (*p* > 0.05) on the embryo production. The average of the total embryos produced by donors with values “A” and “B” was 8.5 ± 3.92, vs. 7.4 ± 5.53 embryos, respectively (*p* > 0.05). Other factors, such as bull used for insemination, year (8.5 ± 5.19 in 2016, 8.5 ± 3.56 in 2017, 7.4 ± 3.81 in 2018, 7.7 ± 6.16 in 2019, and 5.5 ± 6.25 in 2020), or heat stress (8.2 ± 4.88 under heat stress, vs. 7.2 ± 5.28 without heat stress) did not affect the number of total embryos obtained (*p* > 0.05). These factors did not affect the percentage of viable embryos obtained (*p* > 0.05).

The results obtained from the donors’ metabolite assessment at embryo flushing are summarized in Table 1.

The donor plasma metabolites revealed that the concentrations of BHB and TC decreased with the years of study, while glucose and TG increased.

The Pearson correlation test revealed no statistical correlations between the metabolite values of the analyzed donors and their embryo production (total embryos and viable embryos). Very slight but significant correlations were demonstrated between the value of NEFAs and the percentage of degenerated embryo/cells (r = −0.275; *p* = 0.0018).

### 3.2. Embryo Transfers and Recipients’ Pregnancy Results

From the 687 Japanese Black embryos obtained and transferred, a total of 270 were fresh embryos and 417 embryos were transferred after a direct, one-step frozen–thawed procedure. From the fresh transferred embryos, 92 were performed 2018 (92/270; 34.07%), 114 ET, 2019 (114/270; 42.22%) and 64 ET, 2020 (64/270; 23.70%). All transfers of fresh embryos were performed at the Mudéjar-Wagyu farm, on the recipients’ herd previously described. The time distribution of the performed ETs with frozen embryos was the following: 2018: 49/417; 11.75%; 2019: 88/417; 21.10%, 2020: 148/417; 35.49% and 2021: 132/417; 31.65%). A total of 140/417 transferred frozen–thawed embryos were imported and 277 were own embryos obtained from the flushings performed on the Mudéjar-Wagyu farm heifers. Frozen–thawed embryos were randomly transferred at Holstein dairy farms or in the recipients’ herd at the Mudéjar-Wagyu farm.

We found no statistical differences in conception rates (CR) depending on the embryo type (*p* > 0.05): 50.0% CR for fresh embryos (135/270) vs. 47.5% for frozen–thawed ones (198/417), or regarding the origin of the embryos: 48.3% for the own (264/547) vs. 49.3% for the imported embryos (69/140). In the subset of fresh embryos, the embryo quality did not significantly affect CR (*p* > 0.05): 50.9% of CR for embryos type 1 (110/216), 44.0% for type 2 (22/50), and 75.0% for type 3 (3/4).

The factor “farm of recipients” did not significantly affect conception rates (*p* > 0.05) with a CR of 50.6% at the Mudéjar-Wagyu farm (203/401) vs. 44.0% at the external farms (130/286). No statistically significant differences in conception rates were observed by the breed of the recipients (*p* > 0.05). The CR was 51.4% for Simmental (142/276), 53.1% for Charolais (17/32), 41.0% for Brown Swiss (22/41), 52.0% for crossbred recipients (22/52) and 45.5% for Holstein (130/286). Similarly, having suckling or not suckling calves did not reveal a significant effect on CR. Cows with suckling calves obtained 50.0% (50/101) of CR, vs. 51.0% (153/300) for cows without suckling calves (*p* > 0.05).

Regarding the numerical variables measured on the recipients at the ET procedure, BCS, and open days (OD, days from the last calving to ET), none of them differed significantly between pregnant and non-pregnant cows after ET. The BCS of the non-pregnant recipients was 3.7 ± 0.37 vs. 3.7 ± 0.39 for the pregnant ones (*p* = 0.13), and the open days values were 190 ± 109d vs. 196 ± 103d, respectively (*p* > 0.05). However, heat stress did affect conception rate outputs, while donor parity and transfer number matched with numerically different CR values (Table 2).

A total of 44 animals lost pregnancy before day 120 of pregnancy (pregnancy loss rate or PL), which made a PL rate of 13.2% (44/333).

Although the regression model did not show statistical differences in CR values induced by the recipients’ parity and the number of ET, the chi-square analyses did, with a *p*-value of 0.006 for the CR of heifers vs. primiparous and multiparous and the differences between CR after the first ET or further ETs (*p* = 0.04).

A total of 246 ET recipients were blood sampled: 124 pregnant and 122 non-pregnant after ET. Results from the metabolite analyses are summarized in Table 3.

Among the pregnant animals, only the total triglycerides tended to differ between cows that suffered PL after having gotten pregnant (90.0 ± 32.04 mg/L for cows with PL vs. 10.0 ± 42.85 mg/L for those without PL; *p* = 0.14). Finally, the Spearman correlation test demonstrated no statistically significant correlations between any variable.

## 4. Discussion

Our study demonstrates MOET efficiency in a Japanese Black herd in Spain similar to the efficiency in Japanese Black herds elsewhere in the world. Moreover, our data suggest that the efficiency of producing Japanese Black embryos and their quality were not significantly affected by levels of leptin or key plasma metabolites in donors during superovulation, nor were they affected by year, heat stress, bull used, or donor genetics. In contrast, CR after embryo transfer was significantly lower in the presence of heat stress, and it depended on recipient parity and embryo transfer number. Additionally, levels of total cholesterol, LDL, urea, and glucose were higher in pregnant recipients than in non-pregnant cows.

An average of 5.3 ± 4.34 viable embryos per flushing were obtained in the present study, similar to the averages of 6.2 ± 1.2 and 5.1 ± 1.1 reported for Japanese Black cattle in Japan after using two commercial FSH drugs [50]. These averages are also similar to those published for different breeds by the American Embryo Transfer Association based on data from its Annual Survey of Members [51] and averages across the USA published by Phillips et al. [52], which were 7.46 in 2002 (*n* = 170,397 viable embryos), 5.69 in 2007 (*n* = 326,920 viable embryos), and 6.80 in 2007 (*n* = 301,401 viable embryos).

We did not observe a significant influence of donor genetic background or bull on embryo production or quality, yet several studies have pointed out the importance of selecting highly valuable animals as donors for MOET [52,53,54]. In fact, the heritability of the total number of embryos or oocytes is 0.26 in Japanese Black cattle, while the heritability of the total number of grade 1 embryos is 0.17 [55]. Our results may reflect that we excluded from the study donors that produced no embryos, and we used only highly fertile bulls. Whatever the explanation, our results highlight the potential for further genetic improvement of the breed. Our analysis suggests that more valuable Japanese Black heifers can show embryo production and reproductive efficiency similar to those of unselected animals.

We observed that heat stress significantly reduced CR after embryo transfer, consistent with studies on European beef and dairy cattle [36,56,57,58]. Heat stress reduces bovine reproductive efficiency [37]: it reduces the superovulatory response, embryo quality, fertilization rate, and yield of transferable embryos in MOET programs [59]. Heat stress may exert these effects by affecting secretion of reproductive hormones, interfering with embryo development, or inducing oxidative stress in cells [59,60,61,62]. While one study of Japanese Black cows found that heat stress did not significantly affect the quality of aspirated oocytes or their developmental competence during IVF, it may affect follicular recruitment in non-pregnant cows [63]. Our failure to detect a significant influence of heat stress on embryo production may reflect the climate at the study farm, where Temperature-Humidity-Index (THI) values are not so high and air humidity is low, so temperatures are low at night, even in summer. For example, during the last year of the study (2020), maximal and minimal temperatures in July were 30.2 °C, 14.6 °C in July, 29.5 °C, and 14.2 °C in August [42]. Moreover, the animals on the study farm had access to shaded areas and well-ventilated barns.

Donors in our study had an average BCS of 3.5 ± 0.57, and no donor was over–conditioned, implying appropriate nutritional planning that should maximize embryo production. Intermediate BCS of 2.5–4 on a scale from 1 to 5 [49] has been associated with significantly greater embryo production [64]. Conversely, overfeeding leads to high peripheral concentrations of both luteinizing hormone and insulin, which reduce fertilization rate and embryo quality [65], as well as in vivo embryo production [66]. Since nutritional planning can influence donor metabolic status [56], metabolic indicators in our donors were examined closely. Levels of key metabolites did not correlate significantly with embryo productivity, and all biomarkers were within physiological ranges [56,67], further supporting the idea that our donors were in homeostasis. In fact, comparison of metabolic markers over the course of the study suggests that nutritional status improved in the later years, as reflected in lower levels of lipidic biomarkers and leptin as well as higher glucose levels in plasma. Lower lipid metabolism has been linked to better cattle health [68]. The present study found a significant, low intensive, correlation between levels of BHB and the number of unfertilized oocytes. Higher BHB level indicates negative energy balance, which may impair reproductive efficiency [69]. It may be possible to further improve embryo production in the Japanese Black breed by administering rumen bypass polyunsaturated fatty acids, which has been shown to increase the number of transferable embryos after superovulatory treatments [70,71].

In the present study, flushing number appeared to significantly influence the levels of certain metabolites in the donor. This result may actually reflect the influence of dam age: dam age was 18.8 ± 4.30 months for flushings 1–4, but 30.9 ± 4.49 months for flushings greater than 5. Consistent with this idea, Zoda and colleagues [55] reported a decrease in the number of embryos per flushing with increasing age of Japanese Black cows. Those authors attributed this age effect to age-dependent metabolic changes, which have also been reported in dairy cattle [72].

The donor cows in the current study showed substantially lower leptin levels (0.5 ± 0.75 ng/mL) than other Japanese Black cows, such as 4.7–6.5 ng/mL in one study [73] or 2.7 ± 0.41 ng/mL in another [20]. Holstein cows in moderate body condition show even higher leptin levels of 9–10 ng/mL [64]. Leptin regulates ovary function, oocyte maturation, and embryo development before implantation [74]. Leptin also increases embryo quality during superovulation [75], and there appears to be an optimal concentration, below or above which reproductive outcomes worsen [76,77]. These comparisons may not be entirely valid because most of those previous studies examined younger, fattening cattle, which were likely over conditioned. In addition, leptin levels can vary with breed, nutritional management, and body condition [78]. The low leptin levels in the present study probably reflect that the animals were allowed to move freely, and nutrition was strictly controlled to avoid over conditioning. Further studies are needed to clarify the relationship between leptin levels and reproductive outcomes in Japanese Black cattle.

CRs after embryo transfer in our study were 50.0% for fresh embryos and 47.5% for frozen–thawed embryos, very close to the corresponding values of 51.1% and 44.0% published for another herd of Japanese Black cattle [70]. CRs in the current study did not differ significantly across various breeds, including Simmental, Charolais, Brown Swiss, Holstein, or crossbreeds, which is consistent with a report of similar CRs between beef and dairy breeds [79]. The similarity in CR between fresh or frozen–thawed embryos in the Japanese Black cattle in the present study echoes previous results for Aberdeen Angus [80]. In fact, embryo quality appears to be the strongest determinant of CR after embryo transfer [81,82]. Only grade 1 embryos were frozen in our study, in accordance with recommendations [81], while embryos of grades 2–3 were usually transferred freshly, which improves the probability of pregnancy [47,82]. This dual practice of freezing only the best embryos and of freshly transferring lower-quality embryos probably helped close the gap in CR between the two types of transfer, as reported for Aberdeen Angus, Holstein, and crossbreeds [58,80].

CR in the present study was lower among heifers than among primiparous or multiparous cows. This is the opposite of what is generally observed in bovine [83]. The present finding may be explained by careful recipient selection on the Japanese Black farm, such that only highly efficient animals were repeatedly used for embryo transfer. In addition, most of the heifers were from external Holstein farms, where all transfers involved high-quality frozen–thawed embryos. A regression model that included all these factors did not detect a significant effect of parity or its interaction with other factors.

Pregnant recipients in the present study had significantly higher levels of total cholesterol, LDL, glucose, and urea than non-pregnant recipients. Therefore, these levels may serve as metabolic biomarkers to guide recipient selection, given the importance of appropriate energy metabolism for CR in beef cattle [84]. During the first stages of pregnancy, fatty acid degradation and oxidative metabolism may increase and thereby affect the transport of nutrients from the blood into the uterus, ultimately influencing fertility [85,86].

## 5. Conclusions

The results of this study demonstrate adequate non-surgical, in vivo production of Japanese Black embryos of sufficient quality for MOET under Spanish management and environmental conditions. Therefore, good MOET efficiency can be expected with this breed under conditions that may differ substantially from those in Japan. Nutrition and animal welfare should be closely managed in order to maximize the productivity of donor heifer flushing and post-transfer pregnancy rates in recipient animals. Heat stress should be avoided during transfers of Japanese Black embryos. These results provide a useful basis for optimizing Japanese Black breed reproductive management as well as MOET programs in this and other cattle breeds.

## Figures and Tables

**Table 1:** Donor blood plasma metabolites at embryo flushing and factors affecting Japanese Black heifers submitted to a MOET program at a Spanish farm.

		Year				Flushings Order		
Metabolite	Median (Min–Max)	2018	2019	2020	*p*-Value	1 to 4	>4	*p*-Value
BHB (mmol/L)	0.42 (0.06–0.89)	0.80 (0.29–0.89)	0.37 (0.06–0.60)	0.43 (0.28–0.76)	<0.0001			
NEFA (mmol/L)	0.24 (0.06–0.77)							
TC (mg/L)	110.0 (64.0–181.0)	114.0 (101.0–167.0)	134.0 (67.50–181.0)	86.50 (64.0–134.0)	<0.0001	103.60 (74.63–158.28)	133, 25 (121, 30–141, 25)	0.022
HDL (mg/L)	46.0 (19.0–84.0)	55.90 (35.0– 65.70)	54.00 (19.0– 84.0)	32.50 (26.0–43.0)	<0.0001	42.65 (31.50–66.93)	61.43 (52, 39–67, 36)	0.002
LDL (mg/L)	14.0 (3.0–28.0)							
GLU (mg/L)	82.0 (60.0–154.0)	76.0 (62.0–92.0)	80.0 (60.0–93.0)	89.50 (73.0– 154.0)	<0.0001	84.0 (73.0–107.50)	71.69 (68, 25–75, 75)	<0.0001
LAC (mg/L)	19.60 (4.45–87.0)	25.67 (11.27–30.48)	17.0 (4.45–53.80)	20.0 (7.0–87.0)	0.047			
TG (mg/L)	23.0 (14.0–62.0)	23.0 (14.0–29.0)	21.0 (14.0–43.0)	36.0 (22.0–62.0)	<0.0001	26.50 (14.75–45.0)	24.09 (21, 13–27, 31)	0.033
UR (mg/dL)	24.0 (11.0–41.0)							
FRU (mg/L)	275.0 (200.0–508.0)							
LEP (ng/mL)	0.19 (0.01–2.28)	0.11 (0.06–0.27)	0.84 (0.06–1.48)	0.08 (0.01–0.39)	<0.0001	0.29 (0.08–2.28)	0.74 (0, 54–1, 09)	0.016

Abbreviations: BHB (beta hydroxybutyrate; mmol/L), NEFA (non-esterified-fatty acid; mmol/L), TC (plasma total cholesterol; mg/L), HDL (high density lipoprotein cholesterol; mg/L), LDL (low density lipoprotein; mg/L), GLU (glucose; mg/L), LAC (lactate; mg/L), TG (triglycerides; mg/L), UR (urea; mg/dL), FRU (fructosamine; mg/L), and LEP (leptin, ng/mL). * Measurements from 76/137 donors.

**Table 2:** Conception rate after embryo transfer (ET) of Japanese Black bovine embryos by recipients’ parity, transfer number, and heat stress at ET.

		CR (%; *n*/N)	*p*-Value	OR	95% CI
Recipients’ Parity	Heifers	44.3% (148/337)			
	Primiparous	58.3% (67/115)			
	Multiparous	58.8% (118/235)			
ET number	1	47.0% (235/499)			
	>1	52.1% (98/188)			
Heat Stress at ET	Yes	44.3% (47/106)	0.042	2.06	1.026–4.153
	No	49.2% (286/581)			

Abbreviations: CR = conception rate, OR = odds ratio, CI = confidence interval.

**Table 3:** Recipients’ plasma metabolites at embryo transfer (ET) of Japanese Black bovine embryos by the final pregnancy status of the recipients after the ET (pregnant vs. non-pregnant).

Metabolite	Recipient’s Outcome	Concentration Median (Min–Max)	*p*-Value
BHB (mmol/L)	Pregnant	0.38 (0.10–0.58)	0.32
	Non-pregnant	0.39 (0.21–47.0)	
NEFA (mmol/L)	Pregnant	0.19 (0.02–0.97)	0.89
	Non-pregnant	0.18 (0.02–0.86)	
TC (mg/L)	Pregnant	129.0 (53.0–336.0)	0.025
	Non-pregnant	138.0 (68.0–353.0)	
HDL (mg/L)	Pregnant	51.0 (25.0–87.50)	0.33
	Non-pregnant	53.0 (26.0–88.0)	
LDL (mg/L)	Pregnant	17.0 (4.0–43.0)	0.031
	Non-pregnant	20.0 (2.50–40.0)	
GLU (mg/L)	Pregnant	78.0 (41.0–106.0)	0.005
	Non-pregnant	83.0 (47.50–127.0)	
LAC (mg/L)	Pregnant	14.0 (3.50–53.0)	0.93
	Non-pregnant	14.0 (3.0–73.0)	
TG (mg/L)	Pregnant	23.25 (10.0–118.0)	0.113
	Non-pregnant	21.0 (7.50–83.0)	
UR (mg/dL)	Pregnant	19.0 (7.0–49.0)	0.024
	Non-pregnant	22.0 (2.0–49.0)	
FRU (mg/L)	Pregnant	309.5 (197.0–398.0)	0.158
	Non-pregnant	311.0 (246.0–415.0)	

Abbreviations: BHB (beta hydroxybutyrate; mmol/L), NEFA (non-esterified-fatty acid; mmol/L), TC (plasma total cholesterol; mg/L), HDL (high density lipoprotein cholesterol; mg/L), LDL (low density lipoprotein; mg/L), GLU (glucose; mg/L), LAC (lactate; mg/L), TG (triglycerides; mg/L), UR (urea; mg/dL) and FRU (fructosamine; mg/L). *p* values obtained the U test of Mann–Whitney. * Measurements from 246/687 recipients.

## Data Availability

Data is contained within the article.
